# Clinical Presentation, Pathological Spectrum, and Outcomes of Alcoholic Cirrhosis-Related Immunoglobulin A Nephropathy

**DOI:** 10.1016/j.ekir.2024.02.1397

**Published:** 2024-02-16

**Authors:** Charles Ronsin, Pierre Braud, Christine Kandel-Aznar, Amaury Dujardin, Clémence Petit, David Larmet, Claire Garandeau, Clément Deltombe, Alice Le Clech, Claire Leman, Gilles Blancho, Juliet Schurder, Grégoire Couvrat-Desvergnes, Simon Ville

**Affiliations:** 1 Department of Nephrology and Immunology, Centre Hospitalier Universitaire de Nantes, Nantes, France; 2 Department of Nephrology, Dialysis and Transplantation, Departmental Hospital of Vendée, La Roche-sur-Yon, France; 3 Department of Pathology, University Hospital, Nantes, France; 4 Department of Nephrology, Laval Hospital, Laval, France; 5 Department of Nephrology, Saint Nazaire Hospital, Saint Nazaire, France; 6 Department of Nephrology, Broussais Hospital, Saint-Malo, France; 7 Centre de Recherche en Transplantation et Immunologie UMR 1064, Institut National de la Santé et de la Recherche Médicale (INSERM), Université de Nantes, Nantes, France

**Keywords:** cirrhosis, IgA nephropathy, liver disease

## Abstract

**Introduction:**

Immunoglobulin A nephropathy (IgAN) associated with cirrhosis is frequent but often overlooked because it is largely considered silent. Until now, little has been known about their presentation and outcomes.

**Methods:**

We conducted a retrospective multicenter study on patients with kidney biopsy-proven cirrhosis-related IgAN (cirrhosis-IgAN), diagnosed between 2009 and 2022. We mixed them up with 83 primary IgAN (pIgAN) diagnosed during the same period, using a partitioning clustering approach, to determine common clinicopathological profiles.

**Results:**

All the 46 patients with cirrhosis-IgAN had an excessive alcoholic consumption. Clinical presentation was severe with acute kidney injury (AKI) in 79%; alternative causes of AKI was found in 62% of cases. Three clinicopathological clusters were identified as follows: the first one represented chronic involvement, the second one could be assimilated to mild disease, and the third one corresponded to a membranoproliferative glomerulonephritis (MPGN) pattern and was associated with heavy proteinuria and intrinsic AKI (without alternative causes). Whereas the first 2 clusters were equally distributed between pIgAN and cirrhosis-IgAN, the third was more frequent in patients with cirrhosis. The cumulative mortality rate in cirrhosis-IgAN was 26% and 46% at 1-year and 3-years, respectively. Steroid exposure and moderate or severe AKI were associated with higher mortality and steroid exposure was associated with the occurrence of severe infection.

**Conclusion:**

Our results suggest that high AKI incidence is related to extrinsic causes in most cases but can also be driven by IgA-dominant MPGN in a subset of patients. Steroid use was associated with infectious disease and mortality. Further studies are needed to clarify the role of immunosuppressive treatment in cirrhosis-IgAN patients.

## Graphical abstract

**Figure:**
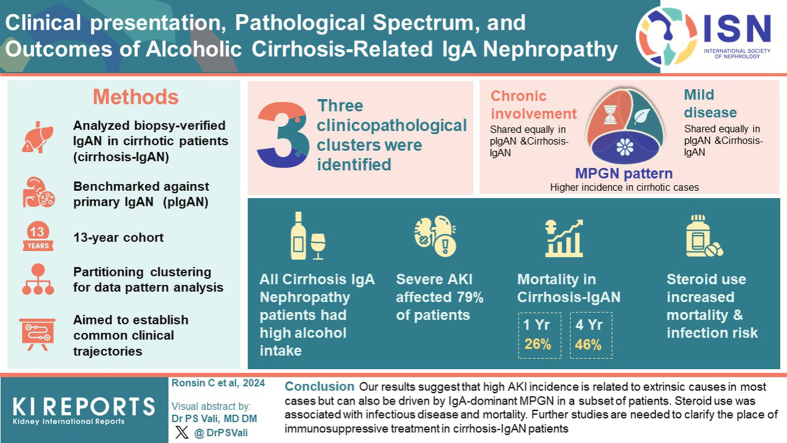

Soon after the first description of IgAN by Berger^1^ in 1967, he and others reported mesangial IgA deposits in kidney biopsies of patients suffering from cirrhosis.^2^^,^^3^ Because IgA deposits were frequently found in autopsy of patients with cirrhosis^2^ with no or mild light microscopy changes and without clinical involvement^4^ mesangial IgA deposit in patients with cirrhosis has long been considered as incidental findings and different from pIgAN.^5^ However, the increased presence of poorly *O*-galactosylated IgA1 in both serum and in glomeruli, which mediate glomerular injury in pIgAN, is found in patients with alcoholic cirrhosis and IgAN associated with cirrhosis.^6^^,^^7^That seemingly common pathogenesis suggest that mesangial IgA deposit in patients with cirrhosis might cause glomerular injury as well.

Although cirrhosis is the most frequent cause of IgAN,^8^ most studies have been limited to pathological analysis, often in the unique setting of liver transplant workup,^9^^,^^10^ with limited clinical data, especially when it comes to evolution. Thus, we conducted a multicenter retrospective study in 5 French hospitals, on patients with liver cirrhosis who underwent a kidney biopsy for urine abnormalities and/or kidney injury that revealed IgAN (cirrhosis-IgAN). Our aims were as follows: to (i) describe initial clinical manifestations, (ii) investigate the different clinicopathologic profiles shared by cirrhosis-IgAN and pIgAN and their respective distribution, and (iii) described the outcomes and renal prognosis factors in patients with cirrhosis-IgAN.

## Methods

### Study Population

Patients diagnosed with IgAN on kidney biopsy and with a history of cirrhosis between January 2009 and September 2022 were identified through pathologist registries and electronic medical records using the keywords “cirrhosis” and “IgA nephropathy” in 5 hospitals located in Western France.

Adult patients were included if they had the following: (i) kidney biopsy-proven IgAN and (ii) cirrhosis defined on liver biopsy, noninvasive measurement of liver stiffness, or on a combination of clinical, imaging, and biological argument (e.g., ascites, beta-gamma bridge on serum electrophoresis, and dysmorphic liver on ultrasound). We also identified 83 patients with pIgAN diagnosed between 2009 and 2022 at Nantes University Hospital to compare them to cirrhosis-IgAN.

This study was approved by the Nantes Hospital General Data Protection Regulation Committee.

### Definition

The decompensated stage of cirrhosis was defined as the past or present occurrence of ascites, hepatic encephalopathy, variceal hemorrhage, or hepatorenal syndrome.^11^ Serum creatinine (ScR) level at diagnosis refers to the highest value within 2 weeks after kidney biopsy. The AKI stage was determined according to the International Club of Ascites classification using the preexisting ScR value.^12^ “Intrinsic” AKI refers to AKI without extrinsic factors explaining the deterioration of renal function (e.g., sepsis or decompensated cirrhosis). Nephrotic syndrome was defined as proteinuria (g/d or g/g) >3 and serum albumin <30 g/l. Estimated glomerular filtration rates (eGFRs) were calculated using the Modification of Diet in Renal Disease 4-variable formula. Baseline eGFRs refer to preexisting GFR values. End-stage renal disease was defined by eGFR <15 ml/min per 1.73 m^2^ or the need for dialysis. Severe infection was defined as any infection requiring hospitalization or extension of hospital length. Patients with available data at month 12 were classified as renal progressors if they reached chronic kidney disease (CKD) stage 4 or 5 and compared with patients with more favorable outcomes at 12 months (nonprogressors).

### Renal Pathology

Renal biopsies were scored according to the Oxford Classification^13^ as follows: mesangial hypercellularity, M0/M1 (if 50% of glomeruli showed >4 mesangial cells/area); endocapillary hypercellularity, E0/E1 (present/absent); segmental glomerulosclerosis, S0/S1 (present/absent); tubular atrophy/interstitial fibrosis, T0/T1/T2 (≤25%, 26%–50%, >50% of cortical area, respectively) and crescents, C0/C1/C2 (no crescents,<25%, ≥25%). In addition, the presence of parietal IgA deposit, duplication of the glomerular basement membrane “double contour,” the percentage of sclerotic glomeruli, presence of interstitial infiltrate, tubular necrosis as well as vascular lesions (ischemic glomeruli, arteriolar hyalinosis, fibro-intimal thickening, thrombotic microangiopathy) were recorded.^14^

### Partitioning Clustering

Partitioning clustering on the pIgAN and cirrhosis-IgAN was performed using a k-means algorithm with k = 3. We chose k = 3, both on the elbow method and after having evaluated the quality of the grouping result. Clustering was based on an unsupervised factor analysis of mixed data, a method of dimension reduction that allows taking into account qualitative variables (mesangial hypercellularity, endocapillary hypercellularity, focal segmental glomerulosclerosis, crescent, interstitial fibrosis >25%, interstitial infiltrate, presence of ischemic glomeruli, arteriolar hyalinosis, fibro-intimal thickening, thrombotic microangiopathy, IgA parietal deposit, and intrinsic AKI occurrence [without confounding factor]) and quantitative variable (urinary protein-to-creatinine ratio and eGFR at baseline). Clusters were derived and visualized with the R package *factoextra* and K-means center values for the 3 groups across the variables were illustrated with bar plots using the R package *ggplot2*.

### Statistical Analysis

Comparisons between discrete variables were made using the Fisher exact test. For continuous variables, comparisons were performed using an unpaired 2-tailed *t*-test. To determine the prognosis factors of IgAN, patients were divided into 2 groups according to their renal outcomes at the end of follow-up. For survival and survival free from severe infection analysis, we performed Kaplan-Meier curves and log-rank test using the R packages *survival* and *survminer*. *P*-values <0.05 were considered significant. RStudio 2022.12 Elsbeth Geranium was used for clustering and all statistical analyses.

## Results

### Clinical Presentation of Cirrhosis Related-IgAN

We retrospectively identified 46 patients with cirrhosis-IgAN biopsied between January 2009 and December 2022. Among the 2 centers with available data, cirrhosis-IgAN accounted for 28 out of 204 cases of IgAN (14%).

Except for 1 trans-jugular biopsy, all patients had percutaneous kidney biopsies, and 2 had severe bleeding (needed red blood cell transfusion). At the time of kidney biopsy, the mean age of patients with cirrhosis-IgAN was 64±10 years, and 8 (17%) were female (Table 1). Causes of cirrhosis were alcohol only in 29 (63%) patients and alcohol plus metabolic (mixed) in the other 17 (37%). Twenty-two patients (48%) had a decompensated cirrhosis; 27 (59%) had a history of hypertension, among them, 17 (37%) patients were treated by angiotensin-converting enzyme inhibitors or angiotensin receptor blockers before kidney biopsy. Fifteen patients (33%) had a history of diabetes and 22 (48%) were tobacco users. Median proteinuria was 2.9 (1.4–4.6) g/g. Complement C3 and C4 serum levels were low in 7 of 34 (21%) and 5 of 34 (15%), respectively. Among the 43 patients with available preexisting ScR, 34 (79%) had AKI, 20 of 34 (58%) had International Club of Ascites stage 3, and 8 patients (17%) needed dialysis. Among patients with AKI (*n* = 34), 21 (62%) had associated conditions (infectious disease, *n* = 9; decompensated cirrhosis, *n* = 6; concomitant sepsis and decompensated cirrhosis, *n* = 2; volume depletion, *n* = 3 [diarrhea, *n* = 2; and rhabdomyolysis, *n* = 1); and recent nephrectomy for renal malignancies shortly before AKI, *n* = 1); whereas the others, 13 (38%) had AKI without alternative causes. Two patients were considered to have *Staphylococcus aureus*–related glomerulonephritis.

**Table 1:** Clinical, biological, and histological characteristics of 46 patients with cirrhosis-related IgAN

Characteristics	Cirrhosis-related IgAN *N* = 46
Clinical features at the time of kidney biopsy	
Age (yr) mean±SD	64 ± 10
Female (%)	8 (17%)
History of hypertension (%)	27 (59%)
History of diabetes (%)	15 (33%)
Tabaco user (%)	22 (48%)
Alcohol abuse (%)	46 (100%)
ACEi or ARB users (%)	17 (37%)
Purpura (%)	11 (24%)
Cirrhosis characteristics at the time of kidney biopsy	
Alcoholic only	29 (63%)
Mixed (metabolic and alcohol)	17 (37%)
History of decompensated cirrhosis	22 (48%)
Ascites	14/22 (64%)
Hepatic encephalopathy	4/22 (18%)
Variceal hemorrhage	8/22 (36%)
Spontaneous bacterial ascites	1/22 (5%)
Laboratory findings at the time of kidney biopsy	
Serum creatinine (mg/dl) mean±SD	3.52 ± 2.54
eGFR (MDRD), mean±SD	36 ± 38
Acute kidney injury (%)^a^	34/43 (79%)
ICA stage	
ICA stage 1	7/34 (21%)
ICA stage 2	7/34 (21%)
ICA stage 3	20/34 (58%)
Alternatives causes of acute kidney injury	21/34 (62%)
Infectious disease	11
Decompensated cirrhosis	8^b^
Hypovolemia	3^c^
Others	1^d^
Dialysis (%)	8 (17%)
uPCR (g/g) median (IQR)	2.9 (1.4–4.6)
uPCR ≥3 g/g (%)	21 (46%)
Nephrotic syndrome	14 (30%)
Microscopic hematuria (%)	39 (85%)
Macroscopic hematuria (%)	8 (17%)
Low C3 (%)	7/34 (21%)
Low C4 (%)	5/34 (15%)
Histopathological characteristics	
Glomeruli (*n*) mean±SD	14 ± 7
Sclerotic glomeruli (%) mean±SD	17 ± 16
MEST-C score, *n*^e^	39
M0/M1, *n*	21/18
E0/E1, *n*	25/14
S0/S1, *n*	25/14
T0/T1/T2, *n*	24/11/4
C0/C1/C2, *n*	34/3/2
Presence of interstitial infiltrate, *n* (%)	25 (54%)
Presence of acute tubular necrosis, *n* (%)	31 (67%)
Vascular lesion, *n* (%)	38 (83%)
Ischemic glomeruli, *n* (%)	19 (41%)
Arteriolar hyalinosis, *n* (%)	19 (41%)
Fibro-intimal thickening, *n* (%)^f^	30/43 (70%)
Thrombotic microangiopathy, *n* (%)	2 (4%)
MPGN pattern, *n* (%)	9 (20%)

ACEi, angiotensin-converting enzyme inhibitor; ARB, angiotensin receptor blocker; eGFR, estimated glomerular filtration rate; ICA, international club of ascites; IgAN, IgA nephropathy; IQR, interquartile range; MDRD, Modification of Diet in Renal Disease equation; MPGN, membranoproliferative glomerulonephritis; uPCR, urinary protein-to-creatinine ratio.

a Three had missing data on baseline serum creatinine, thus, AKI status and ICA stage could not be determined.

b Among the 7 patients with decompensated cirrhosis, 2 had concomitant sepsis.

c Hypovolemia; 2 had diarrhea and 1 had rhabdomyolysis.

d Postnephrectomy for papillary renal cell carcinoma.

e Seven kidney biopsies samples with <8 glomeruli were excluded for the analysis of MEST-C score.

f Three patients did not had arteries on kidney biopsy sample.

Compared to patients with primitive IgAN (*n* = 83), patients with cirrhosis-IgAN were older at presentation (64 vs. 44 years; *P* < 0.001), were more likely to have diabetes (15 [33%] vs. 7 [8%]; *P* = 0.001), or history of arterial hypertension (27 [59%] vs. 30 [36%]; *P* = 0.017) (Supplementary Table S1). At the time of the kidney biopsy, patients with cirrhosis-IgAN had higher ScR values (3.52 ± 2.54 vs. 2.1 ± 1.7; *P* = 0.0016), and proteinuria (3.5 ± 2.5 vs. 2.8 ± 2.29; *P* < 0.001), were more likely to have AKI (34/43 vs. 13/80; *P* < 0.001), and to need dialysis (8 [17%] vs. 4 [5%]; *P* = 0.0264). Although not statistically significant, alternative causes of AKI were found more often in cirrhosis-IgAN (21/34 [62%] vs. 4/13 [31%]; *P* = 0.1).

### The Pathological Spectrum of Cirrhosis Related-IgAN

**Figure 1:**
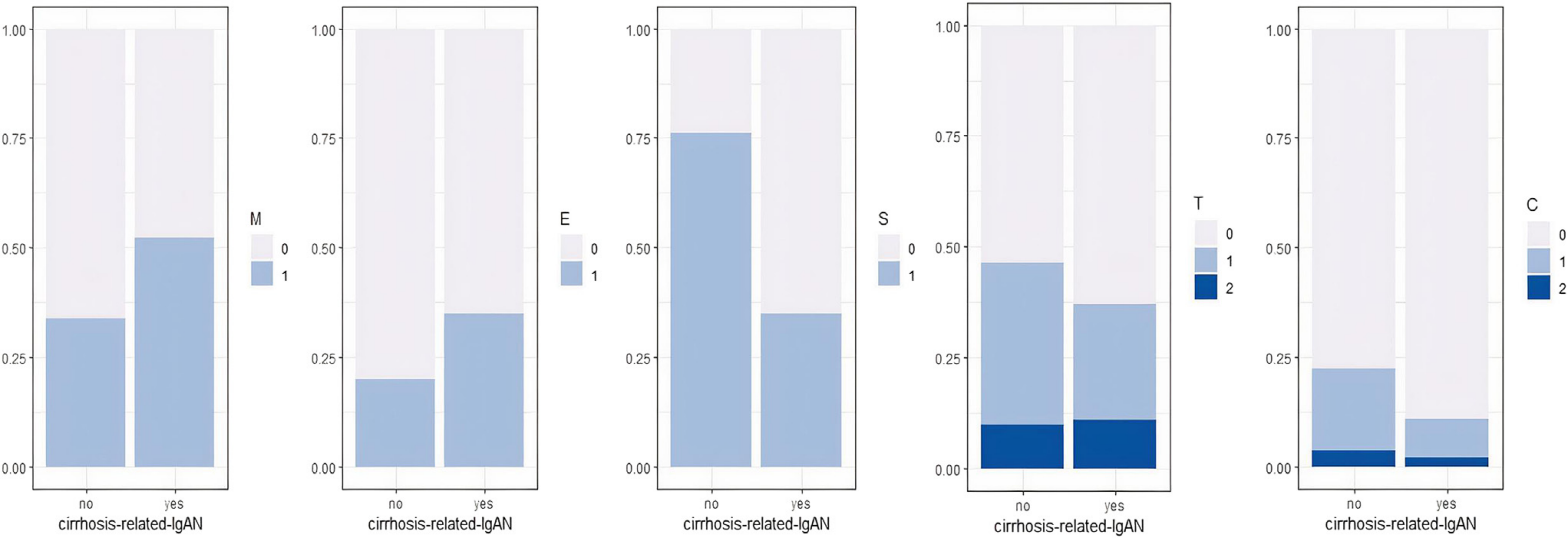
Distribution of the histological variables according to the Oxford classification for cirrhosis-related IgAN and primary IgAN. Kidney biopsy samples with <8 glomeruli were excluded from the analysis. C score, crescent; E score, endocapillary hypercellularity; IgAN, IgA nephropathy; M score, mesangial hypercellularity; S score, segmental glomerulosclerosis; T score tubular atrophy.

Figure 1 represents the distribution of the histological variables according to the Oxford classification for pIgAN and cirrhosis-IgAN. For the latter, the mean number of glomeruli per kidney biopsy was 14±7 with a median (interquartile range) of 13% (5–22)% of sclerotic glomeruli. Seven kidney biopsy samples with <8 glomeruli were excluded from the Oxford analysis. Mesangial hypercellularity (M1) was present in 18 of 39 (46%) biopsies, endocapillary hypercellularity (E1) in 14 of 39 (35%), and focal segmental glomerulosclerosis lesions (S1) in 14 of 39 (35%). The T score was T0 in 24 of 39 (62%), T1 in 11 of 39 (28%), and T2 in 4 of 39 (10%). The C score was C0 in 34 of 39 (87%), C1 in 3 of 39 (8%), and C2 in 2 of 39 (5%) of the biopsies. Compared to primitive-IgAN, there were less segmental glomerulosclerosis (S) (14/39 [36%] vs. 57/71 [80%]; *P* < 0.001), more acute tubular necrosis (31/46 [67%] vs. 26/83 [31%]; *P* < 0.001), more ischemic glomeruli (19/46 [41%] vs. 15/83 [18%]; *P* = 0.0063), and more MPGN pattern (9/46 [20%] vs. 4/83 [5%]; *P* = 0.013) (Supplementary Table S1). Histopathological characteristics were similar whatever the cirrhosis stage, except for the mesangial hypercellularity more frequent in the patients with a history of decompensation (77% vs. 29%; *P* = 0.0014) (Supplementary Table S2).

### Clustering Reveals Distinct Clinicopathologic Profiles

**Figure 2:**
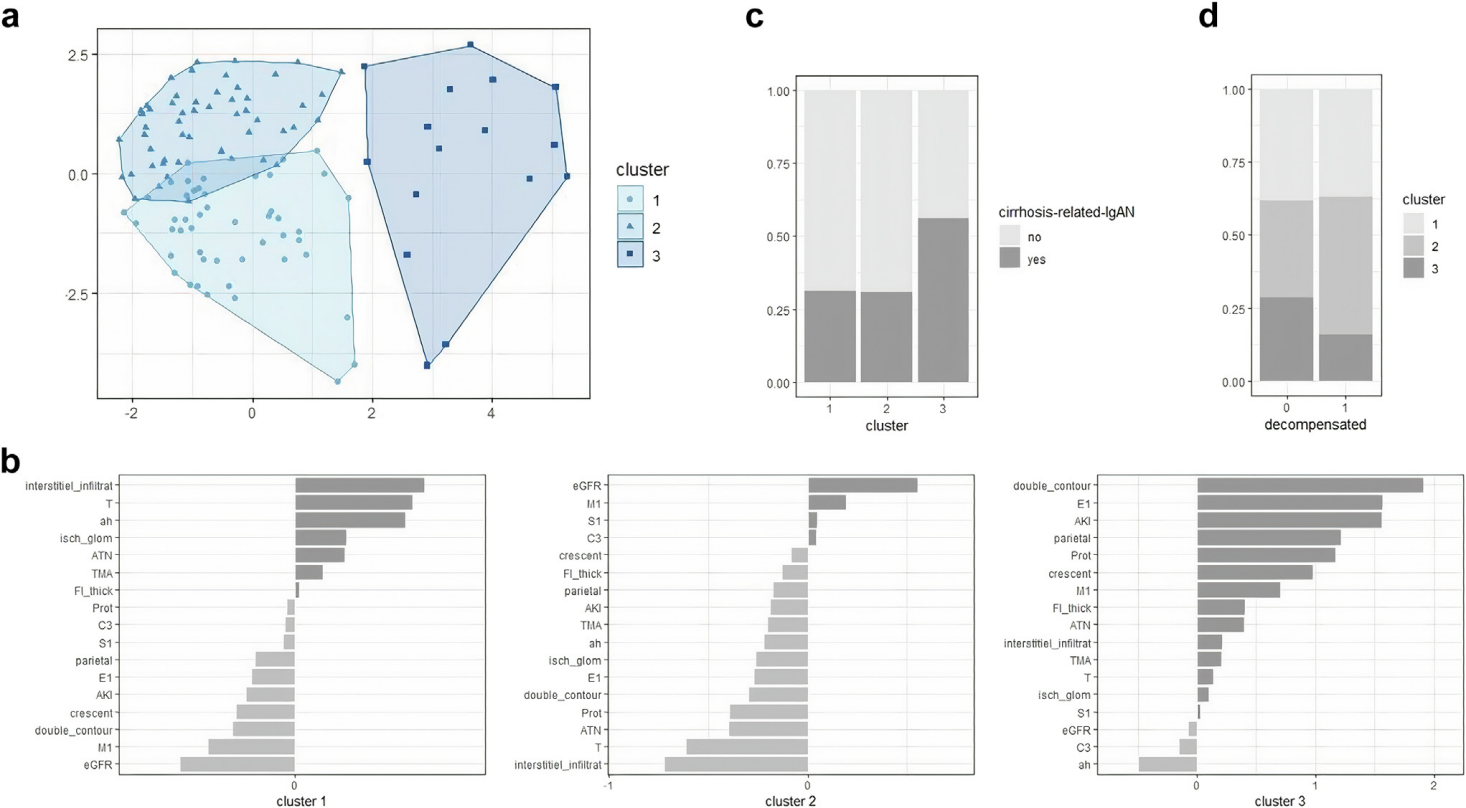
Unsupervised analysis of IgAN (primary IgAN and Cirrhosis-IgAN). (a) Partitional clustering using a k-mean algorithm (k = 3) on our 46 cases of cirrhosis-related IgAN mix-up with 83 biopsy-proved primary IgAN nephropathy diagnosed in the same period. The variables included in the analysis were qualitative variables: mesangial hypercellularity (M1), endocapillary hypercellularity (E1), focal segmental glomerulosclerosis (S1), crescent, interstitial fibrosis >25% (T), interstitial infiltrate, presence of ischemic glomeruli, arteriolar hyalinosis (ah), fibro-intimal thickening (FI_thick), thrombotic microangiopathy (TMA), double contour, acute tubular necrosis (ATN), IgA parietal deposit (parietal) and intrinsic AKI occurrence (without confounding factor); along with clinical quantitative variable: uPCR and preexisting eGFR. (b) Visualization of the K-means center values for the 3 identified clusters across the variables, enabling to annotate them as chronic involvement (cluster 1), mild disease (cluster 2), and MPGN-like pattern (cluster 3) respectively. (c) Frequency of each cluster within the primary IgAN and cirrhosis-related IgAN. (d) Frequency of each cluster in cirrhosis-related IgAN according to the history of decompensated cirrhosis or not. IgAN, IgA nephropathy; MPGN, membranoproliferative glomerulonephritis; uPCR, urinary protein-to-creatinine ratio.

To establish the different clinicopathological profiles and then determine whether some were more frequent in cirrhosis-IgAN, we performed a partitional clustering using a k-mean algorithm on our 46 cases of cirrhosis-IgAN mix-up with 83 biopsy-proved pIgAN diagnosed during the same period. To do so, we considered qualitative variables, mostly histopathological (acute tubular necrosis, mesangial hypercellularity, endocapillary hypercellularity, focal segmental glomerulosclerosis, crescent, interstitial fibrosis >25%, interstitial infiltrate, presence of ischemic glomeruli, arteriolar hyalinosis, fibro-intimal thickening, thrombotic microangiopathy, double contour, C3 deposit, and IgA parietal deposit) and intrinsic AKI occurrence (without confounding factor), along with clinical quantitative variables (urinary protein-to-creatinine ratio and preexisting eGFR). As shown in Figure 2a, we defined 3 distinct clusters. Cluster 1 was annotated as chronic involvement, because the variables that account most for it were interstitial fibrosis >25%, interstitial infiltrate, presence of ischemic glomeruli, and arteriolar hyalinosis, with a negative weight for a high preexisting eGFR (Figure 2b). Cluster 2 was identified as a mild disease, being pushed by mesangial proliferation and high preexisting eGFR with a negative weight for interstitial fibrosis >25% and interstitial infiltrate. Cluster 3 was annotated MPGN-like, because what weighted the most was “double contour,” endocapillary proliferation, parietal deposit, and to a lesser extent, crescent; and for the clinical variables, intrinsic AKI and proteinuria. As shown in Figure 2c, whereas the distribution between cirrhosis-IgAN and pIgAN was similar for clusters 1 and 2, cluster 3 was enriched in cirrhosis-IgAN. However, within the latter, there was no difference in cluster distribution according to the cirrhosis status (i.e., decompensated or not) (Figure 2d). Then we confirmed that in patients with cirrhosis-IgAN, the MPGN pattern, as determined by the pathologist, was more frequent (9/46 [20%] vs. 4/83 [5%]; *P* = 0.013); beyond that, there were more parietal IgA deposits in immunofluorescence when 2+ IgA staining or more were considered (14/40 [35%] vs. 13/83 [16%]; *P* = 0.02) (Supplementary Tables S1 and S3).

### Cirrhosis IgAN With MPGN

Individual baseline features and outcomes of the 9 patients who presented with a cirrhosis-IgAN with an MPGN pattern are detailed in Table 2. Compared to non-MPGN cirrhosis-IgAN (Supplementary Table S4), although not statistically significant, ScR was more elevated (4.8 vs. 3.2; *P* = 0.07), needed more dialysis (44% vs. 11%; *P* = 0.036) and had fewer extrinsic causes of AKI (22% vs. 76%; *P* = 0.013). They had higher values of proteinuria (median, 7.7 vs. 2.2 g/g; *P* = 0.014) and nephrotic syndrome (78% vs. 19%; *P* = 0.0016). Complement C3 was low in 3 of 7 patients (43%) with available test and C4 in 1 of 7 patients (14%) with MPGN. Factor H, factor I and antifactor H antibodies were normal or negative in the 2 patients tested. Factor B antibodies and C3Nef antibodies were negative in the sole patient tested.

**Table 2:** Nine patients with IgA-dominant MPGN pattern associated with cirrhosis

Patients	Age (yr)/Sex	Concomitant or recent infection	Renal features			Immunological findings		Histological features			Treatment	Outcomes/Follow-up
			ScR (mg/dL)	uPCR (g/g)	Hur	C3 g/l (0.9–1.8)	C4 g/l (0.1–0.4)	LM	IF			
									Mg	Cw		
Pt. 1	71/M	No	4	2.2	+	0.89	0.29	MPGN	IgA +++	IgA +++	Steroids	Infectious arthritis leading to the discontinuation of steroids and MMF 2 months after initial KB. ScR 2.22 mg/dl and uPCR 2 g/g/5 mo.
								ATN	C3 +++	C3 +++	MMF	Death (decompensated cirrhosis)
Pt. 2	53/F	No	3.75	3	+	“normal”	0.12	MPGN	IgA +	IgA ++	ACE inhibitor only	ScR 0.96 mg/dl and uPCR 0.4g/g /10 yr
								ATN		C3 +++		
										IgG,IgM traces		
Pt. 3	59/F	Yes, *Staphylococcus aureus* bacteraemia	6.16	8	+	0.55	0.1	MPGN	IgA +++	IgA +++	Steroids	ScR 1mg/dl/5 yr
			Dialysis					Crescent (20%)	C3 +++	C3 +++		
								Severe ATN	IgG,IgM, traces	IgG ++		
								TIN (25%)		C1Q trace		
Pt. 4	73/F	No	5.47	7.68	+	1.11	0.22	MPGN	None	IgA +++	Steroids	Dialysis, death 1 mo after initial KB from sigmoiditis/1 mo
			Dialysis					ATN		C3 ++		
								Crescent (20%)				
Pt. 5	52/M	No	7.96	2.98	+	0.9	0.24	MPGN	IgA +++	IgA +++	Dual blockade of renin- angiotensin system	Dialysis, death 5.7 yr after KB from traffic accident
								ATN	C3 ++	C3 xyd++		
Pt. 6	66/M	No	6.2	9.36	+^a^	NA	NA	MPGN	IgA ++	IgA ++	Steroids 40mg/d	Dialysis, death 2 yr after KB from lung malignancy
			Dialysis					ATN	C3 ++	C3 ++		
									IgM trace			
Pt. 7	62/M	No	1.2	8.97	+	1.25	0.28	MPGN	IgA +++	IgA +++	Steroids 80mg/d	Death 4.5 mo from KB from *Staphylococcus aureus* bacteriemia, ScR 5.1 mg/dl
								ATN	C3 ++	C3 ++	+	
											ACE inhibitor	
Pt. 8	81/M	No	6.25	4	+	NA	NA	MPGN	IgA ++	IgA ++	Steroids	Dialysis, death 11.5 mo after KB from exacerbation of COPD
								Crescent (30%)	C3+	C3 +		
								ATN				
Pt. 9	70/M	No	2.46	13	+	0.85	0.2	MPGN	IgA +++	IgA +++	Steroids	Death 1.6 mo after KB from decompensated cirrhosis, last ScR 1.58 mg/dl, uPCR 4.6 g/g
									C3 +++	C3 +++	+	
									IgM +	IgM +	ACE inhibitor	

ACE, angiotensin-converting enzyme; ATN, acute tubular necrosis; COPD, chronic obstructive pulmonary disease; Cw, capillary wall; F, female; Hur, hematuria; IF, immunofluorescence; KB, kidney biopsy; LM, light microscopy; M, male; Mg, mesangium; MMF, mycophenolate mofetil; MPGN, membranoproliferative glomerulonephritis; Pt., patient; ScR, serum creatinine; uPCR, urinary protein- to- creatinine ratio.

a Macroscopic hematuria.

Only 1 patient (patient 3) had concomitant infectious disease (*S aureus* bacteriemia). None of the 9 patients with IgA MPGN had monoclonal disease or autoimmune disease; and hepatitis B and C virus, serology were negative.

### Management and Outcomes of Cirrhosis-IgAN

**Figure 3:**
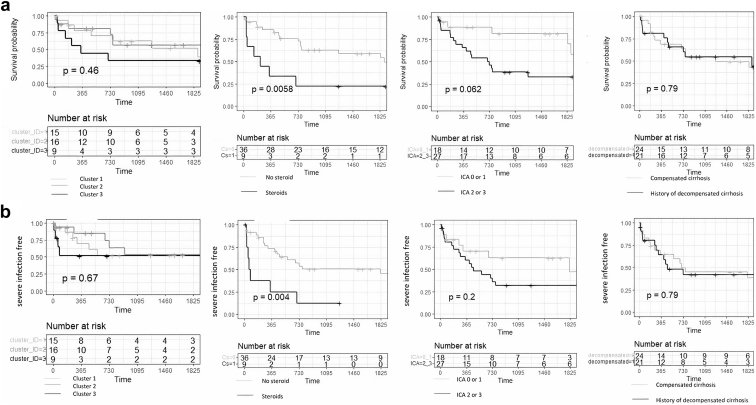
Kaplan-Meier curves of survival (a) and severe infection-free (b) according to the cluster group, steroid use, moderate to severe AKI (ICA stage 2 or 3), history of decompensated cirrhosis or not. The x-axis represents time in days. ICA, International Club of Ascites.

One patient was excluded from the analysis because he was lost to follow-up just after the kidney biopsy. Overall, 27 of 45 patients (60%) had renin-angiotensin system inhibitors (initiated before or after kidney biopsy). Nine (20%) received steroid therapy, 7 (7/9, 78%) in patients within the MPGN pattern, the 2 others had both urinary protein-to-creatinine ratio >3 g/g, with endocapillary hypercellularity in 1 case and >25% of crescentic lesion for the other. There was no difference in the management according to the cirrhosis stage (Supplementary Table S5). Median follow-up time was 25 (5–62) months. The cumulative mortality rates were 0.11, 0.16, 0.26, 0.46, and 0.53 at 3 months, 6 months, 1 year, 3 years, and 5 years, respectively. Steroid exposure was associated with mortality (*P* = 0.006), and although not statistically significant, severe AKI (International Club of Ascites stages 2–3) seemed to be associated with mortality (*P* = 0.06). Regarding mortality, there was no significative difference according to the cluster (chronic involvement/mild disease/MPGN-like) or the cirrhosis status (compensated or not) (Figure 3a). Twenty-one patients (47%) had severe infectious disease during the follow-up and infectious disease was responsible for patients’ death in 7 of 27 cases (26%) (Supplementary Table S6). Severe infection was associated with steroid exposure (*P* = 0.004, Figure 3b).

To further investigate kidney outcomes, for the patients alive and with available data at month-12 postbiopsy, we compared the progressor (i.e., CKD stage 4 or 5, *n* = 8) with the other nonprogressors (*n* = 20). In univariate analysis, factors associated with progressor status were age (67 vs. 59 years; *P* = 0.03), higher ScR (5.1 vs. 2.7; *P* = 0.008), with a trend for the MPGN pattern on kidney biopsy (38% vs. 5%; *P* = 0.058) (Table 3). At the last follow-up, 18 patients (40%) had CKD stage 5, and 7 (16%) had CKD stage 4. In these patients, other factors contributed to the deterioration of renal function were found in 10 of 25 (40%) as follows: liver transplantation (*n* =1), decompensated cirrhosis (*n* = 4), cholesterol emboli (*n* =1), pyelonephritis (*n* =1), septic shock (*n* =2), and urothelial malignancy (*n* = 1) (Supplementary Table S6).

**Table 3:** Prognosis factor of cirrhosis-related IgAN

Characteristics	Progressor *n* = 8	Nonprogressor *n* = 20	*P*-value
Baseline features			
Age (yr) mean±SD	67 ± 9	59 ± 10	0.03
Female (%)	0 (0%)	5 (25%)	0.28
History of decompensated cirrhosis	4 (50%)	10 (50%)	1
Serum creatinine (mg/dl) mean±SD	5.1 ± 1.7	2.7 ± 2	0.008
Acute kidney injury, *n* (%)	7 (88%)	11 (55%)	0.19
Alternative causes of AKI	5 (63%)	9(45%)	0.68
Proteinuria (g/g) median (IQR)	3.5 (2–5.3)	2.8 (1.2–4.3)	0.4
Histological features			
MPGN pattern	3 (38%)	1 (5%)	0.058
M1	5 (63%)	9 (45%)	0.68
E1	3 (38%)	6 (30%)	1
S1	4 (50%)	7 (35%)	0.67
T ≥ 1	4 (50%)	5 (25%)	0.37
C ≥ 1	1 (13%)	3 (15%)	1
Vascular lesion			
Ischemic glomeruli, *n* (%)	5 (63%)	7 (35%)	0.23
Arteriolar hyalinosis, *n* (%)	4 (50%)	7 (35%)	0.67
Fibro-intimal thickening, *n* (%)	6/7 (86%)	14 (70%)	0.63
Thrombotic microangiopathy, *n* (%)	1 (13%)	1 (5%)	0.50
Presence of interstitial inflammation	5 (63%)	12 (60%)	1
Acute tubular necrosis	7 (88%)	13 (65%)	0.37
Management			
Steroids	2 (25%)	2 (10%)	0.55
ACEi or ARB without steroids	4 (50%)	11 (55%)	1

ACEi, angiotensin- converting enzyme inhibitor; AKI, acute kidney injury; ARB, angiotensin receptor blocker; CKD, chronic kidney disease; IgAN, IgA nephropathy; IQR, interquartile range; MPGN, membranoproliferative glomerulonephritis.

Progressor patients was defined as CKD stage 4 or 5 at 12 months.

## Discussion

Our study is the largest involving patients with cirrhosis-IgAN and one of the few to focus on patients with clinical involvement and to further investigate their evolution. We found that alcohol misuse was constant, and clinical presentation was severe with a high incidence of AKI caused either by a concomitant condition or related to an MPGN pattern. Indeed, whereas mild disease and chronic involvement were equally distributed among pIgAN and cirrhosis-IgAN, the latter group was enriched in a cluster identified as MPGN-like that caused nephrotic syndrome along with “intrinsic” AKI (not related to other factors such as sepsis). Cumulative mortality was high, and highly driven by infectious complications, with steroid exposure and AKI at presentation as risk factors. Surprisingly, whether the cirrhosis was compensated or not did not impact the clinical presentation, the histopathologic pattern, or the outcome.

Pathophysiology of IgAN in liver disease is thought to be due to abnormalities of liver clearance of IgA.^15^, ^16^, ^17^ However, IgA 1, whose misdirected secretion by mucosal immune system is the cornerstone of IgAN pathogenesis, including in patients with cirrhosis,^6^ is hardly cleared by the liver.^17^ In pIgAN and in patients with cirrhosis and IgAN, what initiates such a gut-kidney axis is still largely unknown.^18^ Recently, a murine model of alcohol-related liver disease showed a dysfunction of the polymeric IgA transport across the epithelial barrier into the intestinal lumen, leading to an increase of circulating IgA 1.^19^ Currently, whether such a mechanism may explain that all of our patients were alcoholic is speculative, and studies involving nonalcohol patients with cirrhosis and beyond patients with alcohol intoxication without cirrhosis must be conducted before drawing any further conclusions.

The clustering approach method on a dataset made up of cirrhosis-IgAN and primitive IgAN allowed us to determine 3 different clusters, and to find out that the MPGN-like was more frequent in patients with cirrhosis-IgAN. Although the histological MPGN pattern is rarely found in pIgAN, it concerned 20% of patients with cirrhosis-IgAN in our series; and it was associated with massive proteinuria, AKI without confounding factors, and a severe CKD at 12 months. IgA dominant or codominant MPGN pattern had been known since the 1970s in patients with cirrhosis.^3^ However, before 1989 and the hepatitis C virus discovery, confusion with cryoglobulinemia-associated glomerulonephritis could not be ruled out,^3^^,^^20^ because few case reports with such a presentation in alcoholic patients with cirrhosis had been published.^21^ Recently, a retrospective case series of IgA-dominant MPGN was published in which 2 profiles were identified, 1 considered as a rare presentation of pIgAN with a poor prognosis, and another made up of patients with chronic liver disease, for which the authors suspected an infectious trigger.^22^ Our data did not back that hypothesis, because only 1 out of 9 of our patients with the MPGN-like pattern had a concomitant *S aureus* infection. Similarly, among the 37 cases of non-MPGN IgAN cirrhosis, we also identified only 1 case that could be assimilated to a *Staphylococcus* infection-associated glomerulonephritis, suggesting no causal relationship between staphylococcal infections and cirrhosis-IgAN, whatever its presentation. Whereas in patients with the MPGN-like pattern, severe renal impairment seemed driven by the glomerulonephritis itself (intrinsic AKI); in the others, alternative causes promoting an acute tubular necrosis were often present (extrinsic AKI).

Our study is the first to explore outcomes in cirrhosis-IgAN. Evolution was severe with one-quarter of patients dying after 1 year and almost half dying after 3 years. We found that moderate to severe AKI at presentation and steroid exposure were associated with mortality. Furthermore, the renal prognosis was poor because more than half of the patients had ≤30 ml/min per 1.73 m^2^ of eGFR at the last follow-up. We identified several factors associated with poor renal prognosis at month 12 such as age, ScR at baseline, and MPGN pattern at presentation. However, only a few patients were alive with available data at 12 months of follow-up, reducing the reliability of this analysis.

Severe infection was found in almost half of the patients during the follow-up and was one of the main causes of death. Though our study cannot evaluate the efficacy of systemic steroids, there was an association between their use, mortality, and severe infection. This highlights that even when nephrologists observe active renal lesions in patients with cirrhosis-IgAN, especially the MPGN pattern, which was the presentation that triggered the treatment in most cases, they should be aware of a clear risk of severe infections associated with steroids use, whereas its benefit remain speculative. Thus, alternative therapy with a better safety profile must be evaluated. Nefecon, a targeted-release formulation-budesonide, which has proved to be efficient in pIgAN,^23^ could be attractive. However, reduced hepatic metabolism might limit its theoretical benefit by increasing its serum concentration in patients with cirrhosis.^24^ Finally, because numerous therapies aiming to inhibit the different complement pathways are in the pipeline, complement inhibition might be valuable in patients with an aggressive MPGN pattern.^25^

Apart from an immunosuppressive strategy, renal protection is essential in IgAN. Angiotensin-converting enzyme inhibitors and angiotensin receptor blockers are tricky in patients with cirrhosis, particularly at the decompensated stage.^26^ Though not statistically significant, we observed more adverse events (hypotension, AKI, and hyperkalemia) in patients with cirrhosis who are exposed to these drugs compared with those with primitive IgAN. Aldosterone antagonists, which are widely used in patients with cirrhosis and reduce proteinuria,^27^ are certainly a preferred option, because of a less potent hemodynamic effect.^28^ Due to its liver elimination,^29^ uncertainties about the pharmacological profile of SGLT2 inhibitors in patients with cirrhosis persist.

Our study had several limitations inherent to its retrospective design. Although multicentric, few patients were included, a large proportion of missing data on refined parameters restrained the analysis, and no multivariate analysis was possible. Finally, our study is not generalizable to all patients with cirrhosis because percutaneous kidney biopsy is not feasible in patients with advanced cirrhosis and low platelet count. However, this series had the advantage of being pragmatic because all patients underwent kidney biopsies for urine abnormalities or elevated ScR in contrast to several previously published case series of systematic kidney biopsies before liver transplantation.^9^

At a time when the management of IgAN is undergoing a radical transformation, we believe that secondary forms should not be left out. With this perspective in mind, we reported the largest series of cirrhosis IgA-related nephropathy biopsied for cause. The severity of these patients seemed to be linked, for some, to their background and the complications arising therefrom, and for others to a particular form with an MPGN-like pattern for which further investigations are required. Although oral steroid therapy should be avoided because it is associated with a major risk of severe, sometimes fatal, infection, implementation of prospective cohort studies will be needed in the years to come, to clarify the natural history of this disease and assess the risks or benefits of potential therapeutic intervention.

## Disclosure

All the authors declared no competing interests.

## Supplementary Material

Supplementary File (PDF)

**Table S1.** Cirrhosis-related IgA nephropathy versus primitive IgA nephropathy at presentation.

**Table S2.** Clinical, laboratory, and histological characteristics in patients with cirrhosis-related IgA nephropathy according to the history of compensated or decompensated cirrhosis status.

**Table S3.** Immunofluorescence study in patients with cirrhosis-related IgA nephropathy and primitive IgA nephropathy.

**Table S4.** Cirrhosis-associated IgA nephropathy according to the presence of membranoproliferative pattern on light microscopy.

**Table S5.** Treatment and outcomes of patients with cirrhosis-IgA nephropathy according to the history of compensated or decompensated cirrhosis status.

**Table S6.** Management and outcomes of 45 patients with cirrhosis-related IgA nephropathy.
