# Supplementary material for: Clinical Presentation, Pathological Spectrum, and Outcomes of Alcoholic Cirrhosis-Related Immunoglobulin A Nephropathy
Source: Kidney Int Rep. 2024 Feb 16;9(5):1369–78. doi: 10.1016/j.ekir.2024.02.1397 (PMC11069013; doi:10.1016/j.ekir.2024.02.1397)
Supplement: Supplementary File (PDF) [file mmc1.pdf]

Supplemental material

	**Cirrhosis-IgAN N= 46**	**Primary-IgAN N = 83**	**p**
**Clinical features at the time of Kidney biopsy**			
Age (years) mean ±SD	64 ± 10	44 ± 14	**<0.001**
Female (%)	8 (17%)	26 (31%)	0.098
History of hypertension (%)	27 (59%)	30 (36%)	**0.017**
History of diabetes (%)	15 (33%)	7 (8%)	**0.001**
Tabaco user (%)	22 (48%)	28 (34%)	0.13
Alcohol abuse (%)	46 (100%)	5 (6%)	**<0.001**
ACE or ARB users (%)	17 (37%)	23 (28%)	0.32
**Laboratory findings at the time of Kidney biopsy**			
Serum creatinine (mg/dL) mean ±SD	3.52 ±2.54	2.1 ± 1.97	**0.0016**
Acute kidney injury (%)[£]	34/43 (79%)	13/80 (16%)	**<0.001**
Alternatives causes of AKI[$]	21/34 (62%)	4/13 (31%)	0.1
Dialysis (%)	8 (17%)	4 (5%)	**0.0264**
uPCR (g/g) median [IQR]	2.9 [1.4;4.6]	2 [1.3;3.5]	**<0.001**
uPCR ≥ 3 g/g (%)	21 (46%)	28 (34%)	0.19
Macroscopic hematuria (%)	8 (17%)	12 (14.4%)	0.8
Low C3 (%)	7/34 (21%)	1/48 (2%)	**0.0078**
Low C4 (%)	5/34 (15%)	0/48 (0%)	**0.01**
**Histopathological characteristics**			
Glomeruli (n) mean ±SD	14 ± 7	14 +/- 6.9	0.84
Sclerotic glomeruli (%) mean ±SD	17 ± 16	23 +/- 21	0.060
MEST-C score, n (%)	N = 39	N = 71	
M0/M1, n [§]	21/18	46/25	0.3
E0/E1, n	25/14	56/15	0.11
S0/S1, n	25/14	14/57	**<0.001**
T score ≥1, n	15	31	0.69
C score ≥1, n	5	17	0.2
Presence of interstitial infiltrate, n (%)	25 (54%)	42 (51%)	0.72
Presence of acute tubular necrosis, n (%)	31 (67%)	26 (31%)	**<0.001**
Vascular lesion, n (%)	38 (83%)	57 (69%)	0.098
Ischemic glomeruli, n (%)	19 (41%)	15 (18%)	**0.0063**
Arteriolar hyalinosis,n (%)	19 (41%)	38 (46%)	0.7
Fibro-intimal thickening, n (%)*	30/43 (70%)	34/76 (45%)	0.012
TMA, n (%)	2 (4%)	9 (11%)	0.33
MPGN pattern, n (%)	9 (20%)	4 (5%)	**0.013**

Supplemental Table S1. Cirrhosis-IgAN versus primitive IgAN at presentation.

Abbreviation. ACE or ARB, Angiotensin converting enzyme or Angiotensin receptor blockers; uPCR, urinary protein to creatinine ratio; MPGN, membranoproliferative glomerulonephritis.

£)3 patients in the cirrhosis IgAN group and 3 patients in the primitive IgAN group had missing data concerning baseline serum creatinine value.

$) Alternatives causes of AKI in patients with primary IgAN; hearth failure n = 1, pyelonephritis n = 2, Diarrhea and vomiting n =1.

§) MEST-C score was used only in kidney biopsies samples with ≥ 8 glomeruli; 7 were excluded in the cirrhosis-IgAN group and 12 in the primitive IgAN group.

	**Compensated cirrhosis n = 24**	**Decompensated cirrhosis n= 22**	**p**
**Clinical features at kidney biopsy**			
Age – years	63±12	65±8	0.60
Female	7 (29%)	7 (32%)	1
History of hypertension	14 (58%)	13 (59%)	1
History of Diabetes	6 (25%)	9 (41%)	0.23
Causes of cirrhosis			
Alcohol only	13 (54%)	16 (73%)	0.23
Mixed (alcohol and metabolic)	11 (46%)	6 (27%)	0.23
ACE or ARB users	11 (46%)	6 (27%)	0.23
Purpura, n (%)	7 (29%)	4 (18%)	0.496
**Kidney features at kidney biopsy.**			
Serum creatinine	3.5±2.4	3.6± 2.7	0.9
Acute kidney injury	16/21$ (67%)	18 (82%)	0.72
ICA stage 1	3/16 (19%)	4/18 (22%)	
ICA stage 2	2/16 (13%)	5/18 (28%)	
ICA stage 3	11/16 (69%)	9/18 (50%)	
Alternative causes of acute kidney injury	7/16 (44%)	14/18 (78%)	0.076
Dialysis	4 (17%)	4 (18%)	1
uPCR (g/g)	2.2 [1.3;3.8]	3.4 [2.1;4]	0.21
**Histopathological characteristics**			
Glomeruli (n), mean ± SD	14±7	14±8	0.82
Sclerotic glomeruli (%), mean ±SD	17±19	17±13	0.94
Presence of mesangial hypercellularity	7 (29%)	17 (77%)	**0.0014**
Presence of endocapillary hypercellularity	8 (33%)	7 (32%)	1
Presence of FSGS lesion	10 (42%)	6 (27%)	0.36
Presence of crescent	2 (8%)	3 (14%)	0.659
Interstitial fibrosis ≥ 25%	8 (33%)	9 (41%)	0.76
Presence of acute tubular necrosis	16 (67%)	14 (64%)	1
Presence of Interstitial infiltrate	14 (58%)	11 (50%)	0.767
Vascular lesion			
Ischemic glomeruli, n (%)	10 (42%)	9 (41%)	1
Arteriolar hyalinosis,n (%)	10 (42%)	9 (41%)	1
Fibro-intimal thickening, n (%)	16/24 (67%)	14/19 (74%)	0.74
Thrombotic microangiopathy, n (%)	2 (8%)	0 (0%)	0.49
MPGN pattern	6 (25%)	3 (14%)	0.46

Supplemental Table S2. Clinical, laboratory and histological characteristics in patients with cirrhosis-IgAN according to the history of compensated or decompensated cirrhosis status.

Abbreviation. ACE or ARB, Angiotensin converting enzyme or Angiotensin receptor blocker; ICA, international club of ascites; uPCR, urinary protein to creatinine ratio; FSGS, focal segmental glomerulosclerosis; MPGN, membranoproliferative glomerulonephritis.

$) 3 patients had no serum creatinine baseline value.

*) 2 patients in the cirrhosis group and 7 in the primary IgAN group had nor arteries on kidney biopsy sample.

	**Cirrhosis-IgAN N= 46**	**Primitive IgAN N = 83**	**p**
**Mesangial deposit**			
IgA			
Number positive (%)	45 (98%)$	83 (100%)	1
Number ≥ 2+ (%)*	25/40 (63%)	79 (95%)	**<0.001**
IgG			
Number positive (%)	3 (7%)	15 (18%)	0.11
Number ≥2+ (%)*	0/40 (0%)	7 (8%)	0.09
IgM			
Number positive (%)	16 (35%)	51 (61%)	**0.006**
Number ≥2+ (%)*	2/40 (5%)	7 (8%)	0.72
C3			
Number positive (%)	39 (85%)	75 (90%)	0.40
Number ≥2+ (%)*	19/40 (48%)	55 (66%)	0.052
**Glomerular capillary wall deposit**			
IgA			
Number positive (%)	17 (37%)	17 (20%)	0.23
Number ≥ 2+ (%)*	14/40 (35%)	13 (16%)	**0.02**
IgG			
Number positive (%)	3 (7%)	5 (6%)	1
Number ≥2+ (%)*	2/40 (5%)	1 (1%)	0.25
IgM			
Number positive (%)	4 (9%)	6 (7%)	0.74
Number ≥2+ (%)*	0/40 (0%)	3 (4%)	0.55
C3			
Number positive (%)	17 (37%)	12 (14%)	**0.005**
Number ≥2+ (%)*	10/40 (25%)	9 (11%)	0.06

Supplemental Table S3. Immunofluorescence study in patients with cirrhosis-IgAN and primitive IgA nephropathy.

*)No semi quantitative analysis in 6 patients with cirrhosis-IgAN

$) One patient had IgA dominant or co-dominant deposit on glomeruli capillary loop but no mesangial deposit.

	**MPGN pattern N = 9**	**Non MPGN pattern N = 37**	**P**
**Clinical features at the time of kidney biopsy**			
Age (years) mean ±SD	66±10	63±10	0.53
Female (%)	3 (33%)	5 (14%)	0.18
History of hypertension (%)	6 (67%)	21 (57%)	0.72

History of diabetes (%)	3 (33%)	12 (32%)	1
History of decompensated cirrhosis,n (%)	3 (33%)	19 (51%)	0.46
Tabaco user (%)	6 (67%)	16 (43%)	0.28
Purpura (%)	2 (22%)	9 (24%)	1
Decompensated cirrhosis at the time of KB	3 (33%)	10 (27%)	0.70
**Laboratories findings at the time of Kidney biopsy**			
Serum creatinine (mg/dL) mean ±SD	4.8±2.1	3.2±2.6	0.07
Acute kidney injury (%)$	9 (100%)	25/34 (73%)	0.17
Alternative causes of AKI, n (%)	2 (22%)	19/25 (76%)	**0.013**
Dialysis (%)	4 (44%)	4 (11%)	**0.036**
uPCR (g/g) median [IQR]	7.7 [3;9]	2.2 [1.1;3.8]	**0.014**
Nephrotic syndrome	7 (78%)	7 (19%)	**0.0016**
Macroscopic hematuria (%)	2 (22%)	6 (16%)	0.64
Low C3 (%)	3/7 (43%)	4/27 (15%)	0.13
Low C4 (%)	2/7 (29%)	3/27 (11%)	0.27
**Treatment and Outcomes**	N = 9	N = 36$	
ACE or ARB	4 (44%)	23 (64%)	0.45
Steroids	7 (78%)	2 (6%)	**<0.001**
At last follow up			
Follow up (months), median-[IQR]	12 [5-63]	27 [11-62]	0.74
CKD G3a-G5	7 (78%)	30 (83%)	0.65
CKD G5	5 (56%)	13 (36%)	0.24
Severe infections, n (%)	4 (44%)	17 (47%)	1
Death, n (%)	7 (78%)	20 (56%)	0.28

Supplemental Table S4. Cirrhosis associated IgA nephropathy according to the presence of membranoproliferative pattern on light microscopy.

Abbreviation. ACE or ARB, Angiotensin converting enzyme or Angiotensin receptor blockers; uPCR, urinary protein to creatinine ratio; MPGN, membranoproliferative glomerulonephritis.

*) Excluding patients on dialysis.

$) One patient had missing data about treatment and outcome.

	**Compensated Cirrhosis N = 24**	**Decompensated cirrhosis N = 21$**	**p**
Follow up years (months) median-[IQR]	26 [5-56]	24 [12-53]	0.98
**Treatment**			
ACE or ARB	13 (54%)	14 (67%)	0.54
Steroids	5 (21%)	4 (19%)	1
**Outcomes**			
At 12 months	N = 14	N = 14	
Serum creatinine (mg/dL) mean ±SD*	1.4±0.64	1.97±0.96	0.096

eGFR mean ±SD*	68±36	45±24	0.088
CKD G3a-G5	9 (64%)	11 (79%)	0.68
CKD G3a	3 (21%)	2 (14%)	0.53
CKD G3b	2 (14%)	5 (36%)	0.38
CKD G4	1 (7%)	2 (14%)	1
CKD G5	3 (21%)	2 (14%)	1
At last follow up	N = 24	N = 21	
Serum creatinine (mg/dL) mean ±SD	2.3±1.5	2±1	0.5
eGFR mean ±SD	48±33	45±25	0.75
CKD G3a-G5	20 (83%)	17 (81%)	1
CKD G3a	3 (13%)	4 (19%)	0.69
CKD G3b	3 (13%)	2 (10%)	1
CKD G4	3 (13%)	4 (19%)	0.69
CKD G5	11 (45%)	7 (33%)	0.54
Severe infections	9 (38%)	12 (57%)	0.24
Death	16 (67%)	11 (52%)	0.37
Time to death (months), median [IQR]	12 [6-64]	16 [2-25]	0.22

Supplemental Table S5. Treatment and outcomes of patients with cirrhosis-IgAN according to the history of compensated or decompensated cirrhosis status.

Abbreviation. ACE or ARB, Angiotensin converting enzyme or Angiotensin receptor blocker.

$) One patient had missing data about treatment and outcome.

*) excluding patients treated with dialysis

	**Cirrhosis IgAN n =45$**
**Treatment**	
ACE or ARB	27 (60%)
Side effect of ACE or ARB§	5/27 (19%)
Steroids	9 (20%)
Steroids initiated intra-venously.	4 (9%)
**Outcomes**	
Follow-up (month), median [IQR]	25 [5-62]
At 12 months , n	28
Serum creatinine (mg/dL) mean ± SD*	1.7±0.9
eGFR mean ± SD*	55±32
CKD G3a-G5	20 (71%)
CKD G3a	5 (18%)
CKD G3b	7 (25%)
CKD G4	3 (11%)
CKD G5	5 (18%)
At last follow-up	
Serum creatinine (mg/dL) mean ± SD*	2.16±1.3
eGFR mean ± SD*	46±29
CKD G3a-G5	37 (82%)
CKD G3a	7 (16%)
CKD G3b	5 (11%)
CKD G4	7 (16%)
CKD G5	18 (40%)
Severe infections	21 (47%)

Death	27 (60%)
Time to death (months) median [IQR]	15 [5-57]
Causes of death	
Infections	7 (26%)
Decompensated cirrhosis	10 (37%)[£]
Malignancies	6 (22%)
Others	3 (11%)
Unknow	1 (4%)

Supplemental Table S6. Management and outcomes of 45 patients with Cirrhosis-IgAN.

Abbreviation. ACE or ARB, Angiotensin converting enzyme or Angiotensin receptor blocker; eGFR, estimated glomerular filtration rate according to modification of diet in renal disease equation (MDRD).

$) One patient had missing data about treatment and outcome.

§) acute kidney injury n = 2, arterial hypotension n = 2, hyperkalemia n =1.

*) excluding patients treated with dialysis

£) Patients which had decompensated cirrhosis induced by sepsis with further control of the sepsis were classified in “decompensated cirrhosis” group.
